# A case of carotid-axillary bypass for subclavian steal syndrome in an 83-year-old female undergoing hemodialysis

**DOI:** 10.1016/j.ijscr.2023.108974

**Published:** 2023-10-25

**Authors:** Kazunori Hashimoto, Takuya Kawahara, Kosuke Miyoshi, Tetsuya Sato, Satoshi Itoh

**Affiliations:** Department of Cardiovascular Surgery, Yokohama City Minato Red Cross Hospital, 3-12-1 Shinyamashita Naka-ku, Yokohama, Kanagawa 231-8682, Japan

**Keywords:** Carotid-axillary bypass, Subclavian steal syndrome, Hemodialysis

## Abstract

**Introduction:**

Patients undergoing hemodialysis exhibit a high incidence of subclavian steal syndrome. Many cases of endovascular treatment for subclavian artery stenosis were only reported recently; however, the long-term results of surgical treatment are also important. Herein, we report a case of subclavian steal syndrome treated with common carotid-axillary bypass surgery in a patient undergoing hemodialysis.

**Presentation of case:**

An 83-year-old woman experienced dizziness and pain in her left hand during hemodialysis. Computed tomography and angiography revealed severe stenosis and calcified lesions in the left subclavian artery. Ultrasonography revealed a retrograde blood flow waveform in the left vertebral artery. The patient was diagnosed with subclavian steal syndrome. We performed common carotid-axillary bypass for lesions that were difficult to revascularize via endovascular therapy. The post-operative course was uneventful, and the dizziness and numbness in the patient's left hand during dialysis disappeared. Post-operative ultrasonography revealed an antegrade blood flow waveform in the left vertebral artery.

**Discussion:**

Subclavian steal syndrome is an indication for revascularization in symptomatic patients. Endovascular treatment should be considered the first choice; however, surgery should be considered for patients in whom endovascular treatment is difficult, such as those with severe calcification. We chose common carotid-axillary artery bypass because the subclavian approach is a more familiar technique. Until 1 year post-operatively, the patient had not experienced any symptom recurrence, and the shunt flow was well maintained.

**Conclusion:**

Common carotid-axillary bypass can be useful for revascularization of lesions for which endovascular therapy is considered difficult in patients with subclavian steal syndrome.

## Highlights

• Patients undergoing hemodialysis have a high incidence of subclavian steal syndrome.
• This complication may be caused by excessive blood flow due to dialysis access.
• Whether it is due to subclavian artery stenosis or shunt flow needs to be determined.
• Common carotid-axillary bypass can be used for revascularization of lesions.
• For 1 year, the patient did not experience recurrence and shunt flow was maintained.

## 1 Introduction

Subclavian steal syndrome is a pathological condition in which reflux from the ipsilateral vertebral artery occurs due to subclavian artery stenosis or occlusion, thereby resulting in basilar artery insufficiency. The reported prevalence of subclavian steal syndrome ranges from 0.6 to 6.4 % [1]. Patients undergoing hemodialysis have a high incidence of subclavian steal syndrome, which may be caused by excessive blood flow due to dialysis access [2,3]. Many cases of endovascular treatment for subclavian artery stenosis have been reported in recent years; however, the long-term results of surgical treatment are also important. In this paper, we report a case of subclavian steal syndrome treated with common carotid-axillary bypass surgery in a patient undergoing hemodialysis.

## 2 Presentation of case

**Fig. 1:**
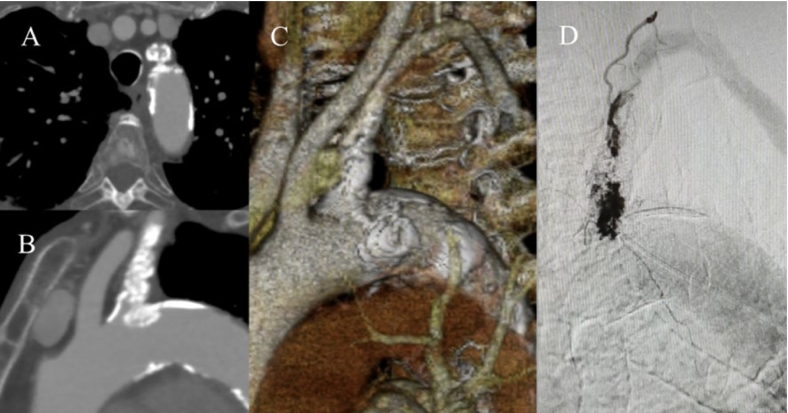
Pre-operative computed tomography findings. The axial (A), sagittal (B), and three-dimensional images (C) reveal a severely stenotic lesion with calcification of the left subclavian artery. The pre-operative angiography image reveals that the axillary artery drains via the collateral circulation (D).

The case was reported in line with the SCARE criteria [4]. An 83-year-old woman was admitted to our hospital for dizziness and pain in her left hand during hemodialysis. She developed chronic kidney failure due to polycystic kidney disease and required dialysis three times weekly. A detailed examination was performed as removing the blood during hemodialysis would have been difficult. Ultrasonography revealed a decrease in the shunt flow volume to 219 mL/min and stenosis of the basilic vein in the outflow tract. The blood pressure in the patient's left arm was 30 mmHg lower than that in the right arm due to the left subclavian artery stenosis. Computed tomography revealed a severely stenotic lesion with calcification of the left subclavian artery (Fig. 1A, B, C). The pre-operative angiography image revealed that the axillary artery drained via the collateral circulation (Fig. 1D). Ultrasonography revealed a retrograde blood flow waveform in the left vertebral artery, and magnetic resonance imaging revealed no significant stenosis in the cervical and intracranial arteries; therefore, subclavian steal syndrome was suspected. Subclavian steal syndrome may be aggravated by previous treatment of outflow tract stenotic lesions.

**Fig. 2:**
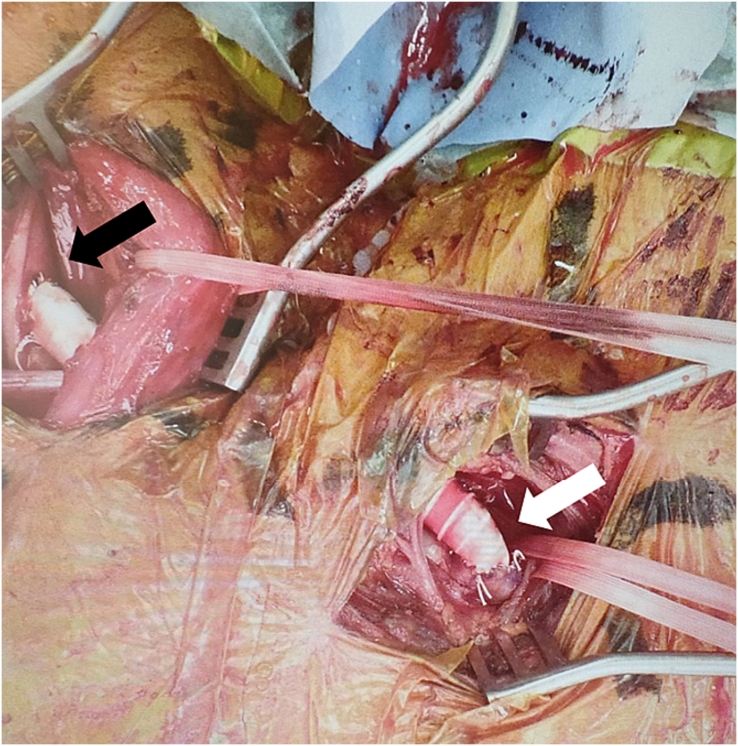
Intra-operative photograph of the polytetrafluoroethylene graft after anastomosis. The image shows the proximal anastomosis to the common carotid artery (black arrow) and the distal anastomosis to the axillary artery (white arrow).

We chose to surgically treat the left upper limb ischemia under general anesthesia. The left common carotid artery was exposed through a longitudinal incision along the medial aspect of the left sternocleidomastoid, and the left axillary artery was exposed through an infraclavicular incision. An 8-mm ringed polytetrafluoroethylene graft (Gore PROPATEN graft; W. L. Gore & Associates, Inc., Newark, DE, USA) was tunneled under the sternocleidomastoid and clavicle. After linearly incising the left common carotid artery, an end-to-side proximal anastomosis was created. The graft was routed through the root and distally anastomosed to the axillary artery in an end-to side manner (Fig. 2). After reperfusion, brachial artery pulsation was palpable. The occlusion time of the left common carotid artery was 12 min, during which no decrease in cerebral oxygen saturation was observed.

**Fig. 3:**
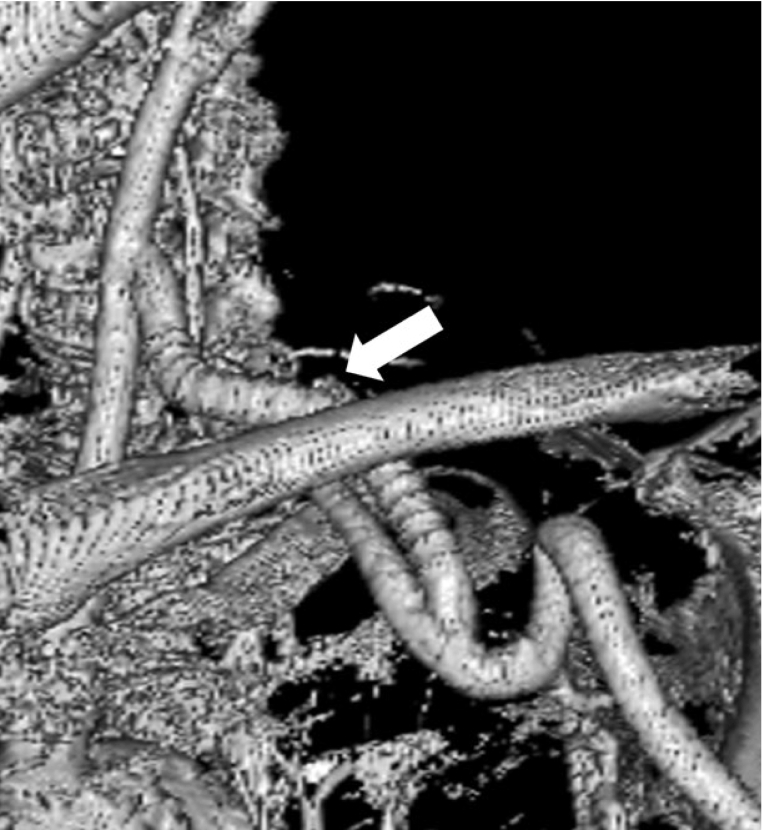
Three-dimensional computed tomography images 1 week post-operatively. The bypass graft is patent (white arrow).

**Fig. 4:**
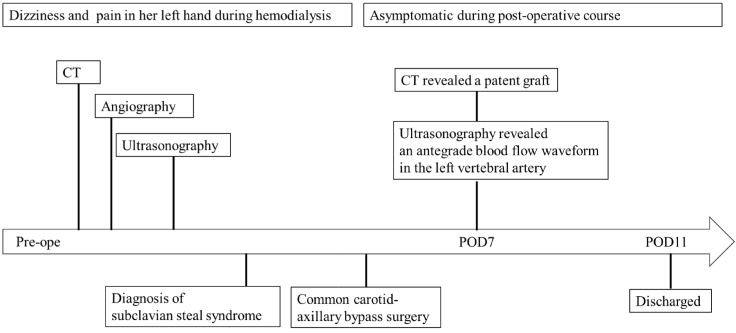
Timeline of diagnostic assessment, treatment and outcomes.

The patient was asymptomatic during post-operative hemodialysis. Computed tomography 1 week post-operatively revealed a patent graft (Fig. 3), and ultrasonography simultaneously revealed an antegrade blood flow waveform in the left vertebral artery. The patient was discharged on post-operative day 11. The stenotic lesion of the basilic vein in the outflow tract was later treated, and the shunt flow volume improved to 1070 mL/min. Until 1 year post-operatively, the patient had not experienced any recurrence of symptoms, and the shunt flow was well maintained. The timeline of the diagnostic evaluation, surgical procedure and outcome is described in Fig. 4.

## 3 Discussion

Symptomatic subclavian artery steal syndrome in limbs with shunts may be caused by subclavian artery stenosis or high-flow shunts. Excess blood flow in the shunt causes vertebrobasilar artery insufficiency, and patients without subclavian artery stenosis may develop symptoms of subclavian artery steal [5,6].

Subclavian steal syndrome is an indication for revascularization in symptomatic patients. In this case, dizziness and vertebral artery reflux were believed to have occurred owing to subclavian artery stenosis and an arteriovenous fistula. Shunt banding, closure, and addition to the contralateral limb are the treatment options for subclavian artery steal syndrome. Regarding shunt blood flow, blood flow ≥2000 mL/min is considered excess blood flow [5]; however, in this patient, the pre-operative shunt blood flow was low (219 mL/min). Therefore, shunt closure would not have improved the symptoms of subclavian steal. Bavare et al. [7] reported a mean flow volume of approximately 1149 mL/min in an access arteriovenous graft without dialysis-related steal symptoms.

Regarding access expansion in the contralateral upper extremity, we decided to use the existing shunt as a permanent indwelling catheter may have been required due to the lack of autologous veins. If percutaneous transluminal angioplasty is performed to treat stenosis of the flexural cutaneous vein of the shunt outflow before revascularization, an increase in shunt blood flow may exacerbate steal syndrome.

While revascularization by endovascular treatment should be considered the first choice, surgery should be considered for patients in whom endovascular treatment is difficult, such as those with severe calcification. Barakat et al. [8] reported a case in which bypass was performed for subclavian artery occlusion because revascularization through endovascular treatment was difficult.

Axillary-axillary artery bypass, common carotid-subclavian artery bypass, and common carotid-axillary artery bypass are surgical treatment options for symptomatic steal syndrome. Many reports exist regarding common carotid-subclavian artery bypass; however, the supraclavicular approach is associated with problems such as phrenic neuropathy and lymphorrhea [8,9]. Bypass surgery is considered a low-risk surgical procedure for symptomatic patients if the donor carotid arteries do not have atherosclerotic lesions. Furthermore, we selected common carotid-axillary artery bypass as the subclavian approach is a more familiar technique. Favorable long-term results have been reported for bypasses using the common carotid artery as the inflow; the primary patency rate for common carotid-axillary artery bypass surgery was 96 % at 47 months in one report [10]. Axillary-axillary artery bypass surgery is less invasive than bypass with carotid artery inflow; however, the primary patency rate at 5 years is reported to be 76 %, which is lower than that of other bypass procedures [11]. The number of cases demonstrating the long-term results of bypass surgery is limited; therefore, we hope that more cases will be accumulated with positive results reported in the future.

## 4 Conclusion

In this report, we present the case of a patient undergoing hemodialysis who experienced subclavian steal syndrome and underwent carotid-axillary bypass using a polytetrafluoroethylene graft. Common carotid-axillary bypass can be useful for revascularization of lesions for which endovascular therapy is considered difficult. It should thus be determined whether symptomatic subclavian steal syndrome in patients undergoing dialysis is due to subclavian artery stenosis or shunt flow. The indications for revascularization should also be considered. The findings of this case suggest that early diagnosis and appropriate treatment of subclavian steal syndrome are important in patients undergoing hemodialysis.

## Consent

Written informed consent was obtained from the patient for publication of this case report and accompanying images. A copy of the written consent is available for review by the Editor-in-Chief of this journal on request.

## Ethical approval

As case reports that do not contain personally identifiable information do not fall under the category of “research,” our ethics review board has deemed that in-hospital ethical review for case reports is not necessary.

## Funding

This research did not receive any specific grant from funding agencies in the public, commercial, or not-for-profit sectors.

## Author contribution

Kazunori Hashimoto: Data curation, Writing- Original draft preparation, Visualization.

Takuya Kawahara: Data curation, Writing - Review & Editing.

Kosuke Miyoshi: Data curation, Writing - Review & Editing.

Tetsuya Sato: Supervision, Writing - Review & Editing.

Satoshi Itoh: Supervision, Writing - Review & Editing.

## Guarantor

Kazunori Hashimoto will be the guarantor for this paper.

## Research registration number

Not applicable.

## Conflict of interest statement

None.

## References

[1] Osiro S., Zurada A., Gielecki J., Shoja M.M., Tubbs R.S., Loukas M. (2012). A review of subclavian steal syndrome with clinical correlation. Med. Sci. Monit..

[2] Agarwal S., Schwartz L., Kwon P., Selas G., Farkas J., Arcot K. (2018). Subclavian steal syndrome due to dialysis fistula corrected with subclavian artery stenting. Neurol. Clin. Pract..

[3] Cwinn M., Nagpal S., Jetty P. (2017). Subclavian steal syndrome without subclavian stenosis. J. Vasc. Surg. Cases Innov. Tech..

[4] Agha R.A., Franchi T., Sohrabi C., Mathew G., Kirwan A., Thomas A. (2020). The SCARE 2020 guideline: updating consensus surgical CAse REport (SCARE) guidelines. Int. J. Surg..

[5] Schenk W.G. (2001). Subclavian steal syndrome from high-output brachiocephalic arteriovenous fistula: a previously undescribed complication of dialysis access. J. Vasc. Surg..

[6] Kargiotis O., Siahos S., Safouris A., Feleskouras A., Magoufis G., Tsivgoulis G. (2016). Subclavian steal syndrome with or without arterial stenosis: a review. J. Neuroimaging.

[7] Bavare C.S., Bismuth J., El-Sayed H.F., Huynh T.T., Peden E.K., Davies M.G. (2013). Volume flow measurements in arteriovenous dialysis access in patients with and without steal syndrome. Int. J. Vasc. Med..

[8] Barakat T.I., Kenny L., Khout H., Timmons G., Bhattacharya V. (2011). Carotid axillary bypass in a patient with blocked subclavian stents: a case report. J. Med. Case Rep..

[9] Al-Jundi W., Saleh A., Lawrence K., Choksy S. (2009). A case report of coronary-subclavian steal syndrome treated with carotid to axillary artery bypass. Case Rep. Med..

[10] Criado F.J., Queral L.A. (1995). Carotid-axillary artery bypass: a ten-year experience. J. Vasc. Surg..

[11] Huijben M., Meershoek A.J.A., de Borst G.J., Toorop R.J. (2021). Long-term outcome of axillo-axillary bypass in patients with subclavian or innominate artery stenosis. Ann. Vasc. Surg..

